# Inflammatory cytokines mediate thoracic aortic aneurysm formation via plasma metabolites: A two-step Mendelian randomization and single cell sequencing-based investigation

**DOI:** 10.1097/MD.0000000000047561

**Published:** 2026-02-06

**Authors:** Yueheng Liu, Rui Tang, Yitan Fang, Fengming Tian, Tianxiang Gu

**Affiliations:** a Department of Cardiac Surgery, First Affiliated Hospital, China Medical University, Shenyang, Liaoning, China.

**Keywords:** Mendelian randomization, single cell analysis, thoracic aortic aneurysm

## Abstract

Thoracic aortic aneurysm (TAA) is a life-threatening condition characterized by pathological dilation of the aorta. While inflammatory responses have been implicated in TAA pathogenesis, the causal relationships remain elusive. This study aimed to elucidate potential causal associations between inflammatory cytokines, plasma metabolites, and TAA risk using Mendelian randomization (MR) analysis. We conducted bidirectional two-sample MR analysis utilizing genome-wide association study data from 91 inflammatory cytokines (n = 14,824), 1400 plasma metabolites (n = 8299), and TAA (n = 385,857). The inverse-variance weighted method served as the primary analytical approach, with comprehensive sensitivity analyses performed to assess pleiotropy and heterogeneity. Two-step MR analysis was employed to explore potential mediating roles of plasma metabolites. Single-cell sequencing analysis was utilized to detect cell type enrichment and elucidate cellular functions of identified cytokines. Additionally, we conducted an analysis to identify druggable proteins as potential therapeutic targets for TAA. MR analysis revealed that genetically-determined increases in C-X-C motif chemokine 10 (CXCL10) (odds ratios [OR] = 1.149, 95% confidence interval [CI]: 1.009–1.309, *P* = .037) and fibroblast growth factor 5 (OR = 1.101, 95% CI: 1.013–1.196, *P* = .024) were associated with elevated TAA risk. Conversely, C-C motif chemokine 20 (CCL20) (OR = 0.870, 95% CI: 0.759–0.996, *P* = .043) and CD40L receptor (CD40) (OR = 0.906, 95% CI: 0.827–0.992, *P* = .033) demonstrated inverse associations with TAA risk. Two-step MR analysis identified potential mediating metabolites: the phosphate to linoleoyl-arachidonoyl-glycerol ratio for CXCL10, thyroxine and X-24585 for FGF-5, and the creatine to carnitine ratio for CCL20. Single-cell sequencing analysis revealed enrichment of these cytokines in specific cell types and pathways relevant to TAA pathogenesis. Drug–gene interaction analysis identified CXCL10, CCL20, and CD40 as potential targets for treatment of TAA. This study provides robust genetic evidence supporting causal relationships between specific inflammatory cytokines and TAA risk, with plasma metabolites potentially mediating these effects. CXCL10 and FGF-5 were identified as potential risk factors, while CCL20 and CD40 may confer protective effects. These findings offer novel insights into TAA pathogenesis and suggest potential targets for intervention. Further research is warranted to elucidate the underlying mechanisms and validate these results across diverse populations.

## 1. Introduction

Thoracic aortic aneurysm (TAA) is a life-threatening condition characterized by progressive pathological dilation of the aorta, extending from the ventriculo-aortic junction to the diaphragm. This dilation can lead to compression of surrounding organs, aortic dissection, rupture or sudden death.^[1,2]^ TAAs encompass a spectrum of pathologies resulting from complex alterations in the cellular and extracellular milieu, including atherosclerosis, smooth muscle cell dysfunction, extracellular matrix remodeling, and degradation of elastic fibers.^[3]^ Recent research has implicated inflammatory responses in aortic aneurysm formation.^[4]^

A growing body of evidence suggests that inflammatory processes play a pivotal role in TAA pathogenesis. Letizia Scola et al demonstrated that elevated serum levels of interleukin-6 (IL-6) and IL-1β correlate with TAA tissue alterations, such as elastic fragmentation, medial cell apoptosis, and cystic medial changes, as well as immune cell infiltration in aortic aneurysm tissues.^[5]^ Additional research has indicated that inflammatory cell infiltration contributes to TAA pathogenesis.^[6]^ Recent studies have shown that activation of the IL-6/STAT3 signaling pathway contributes to aneurysmal dilation through increased MMP-9 activity, exacerbating extracellular matrix degradation.^[7]^ Furthermore, increased IL-1β levels have been associated with vascular inflammation and senescence in human and mouse TAA samples. Genetic knockout of IL-1β or pharmacological inhibition of IL-1β signaling with a receptor antagonist mitigated vascular inflammation and TAA formation in mice.^[8]^ Despite of these correlations between TAA and inflammatory cytokines, it remains to be determined whether systemic inflammation is a cause of TAA or a consequence of disease progression.

Plasma metabolites, including amino acids, carbohydrates, lipids, and nucleotides, are small molecules originating from cells, tissues, and biological fluids. These molecules have been frequently employed to investigate physiological and pathophysiological processes.^[9,10]^ Raman microspectroscopy analysis revealed alterations in the expression of phenylalanine, tyrosine, tryptophan, cysteine, aspartate, and glutamate in ascending thoracic aortic aneurysm tissue.^[11]^ Changes in serum levels of taurine, histidine, and L-citrulline have been correlated with Marfan syndrome, a condition characterized by aortic root aneurysm formation.^[12,13]^ While these studies have investigated correlations between TAA and inflammatory cytokines or metabolites, the observed associations may be influenced by various biases, including small sample sizes, potential confounders, and reverse causality.

Mendelian randomization (MR) is a statistical approach that employs genetic variations as instrumental variables (IVs) to examine and establish causal relationships in nonexperimental data. This method is based on Mendel law of inheritance, which posits that genetic alleles are randomly allocated and independently transmitted from parents to offspring during gamete formation. This principle ensures that the genetic variants used as IVs are not influenced by external confounders, thereby enhancing the ability to establish causal relationships.^[14]^

Single-cell RNA sequencing technology provides a powerful tool for validating and extending the results of MR analysis. While MR analysis reveals potential causal relationships between certain inflammatory factors and TAA risk, single-cell RNA sequencing enables verification of these findings at the cellular level and offers more detailed information about disease mechanisms. Through single-cell analysis, we can identify in which specific cell types the key genes discovered in MR studies are expressed, and how their expression patterns change between normal and disease states. This cell-specific information helps elucidate the precise roles of these genes in the development of TAA, thereby providing biological context and mechanistic explanations for the MR analysis results.

In this comprehensive study, we conducted a bidirectional two-sample MR analysis to explore causal relationships between 91 circulating inflammatory cytokines and TAA. Additionally, we applied two-step MR analysis to investigate the role of 1091 plasma metabolites and 309 metabolite ratios in mediating the relationship between inflammatory cytokines and TAA. To further elucidate the cellular context of our findings, we employed single-cell sequencing analysis to detect cell type-specific enrichment and elucidate the roles of the identified cytokines in cellular functions and pathways. Lastly, we performed an analysis to identify druggable proteins, exploring their potential as therapeutic targets for TAA. The findings from our in-depth investigation are intended to offer crucial insights and pave the way for precision-oriented therapeutic approaches for TAA in the coming years.

## 2. Materials and methods

### 2.1. Study design

**Figure 1.**
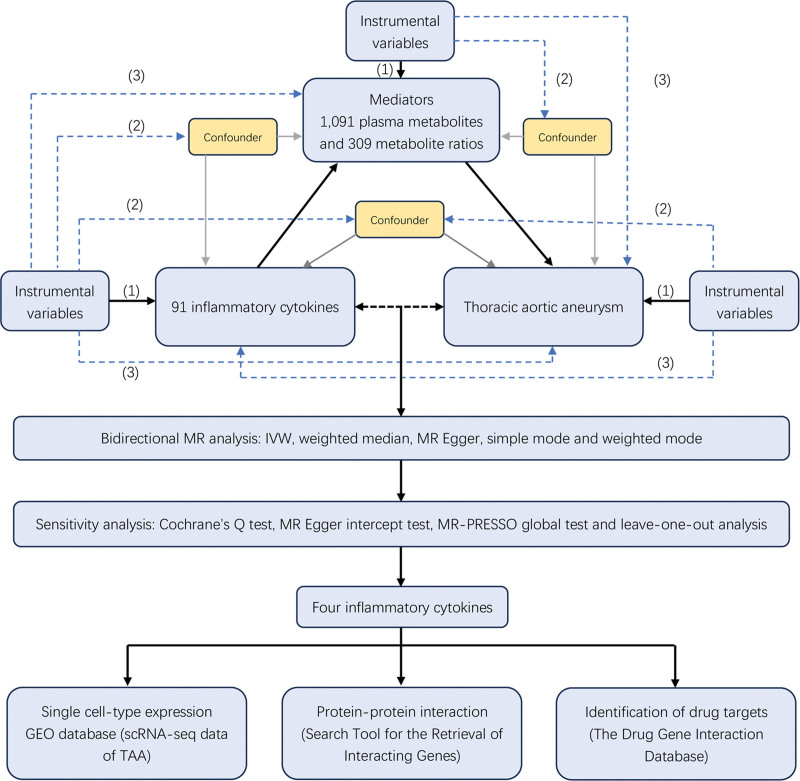
Flowchart overview of the study design.

An overview of the study design is presented in Figure 1. We employed a two-sample bidirectional MR analysis to elucidate the causal relationship between inflammatory cytokines and TAA. To identify potential mediating plasma metabolites, we further conducted a two-step MR analysis.

The genetic variants used as IVs should satisfy 3 core assumptions: strong correlation with the exposure (relevance assumption); independence from confounding factors associated with exposure and outcome (independence assumption); and influence on the outcome solely through the exposure (exclusion restriction assumption).^[15]^ The latter 2 assumptions, collectively referred to as the assumption of no horizontal pleiotropy, can be indirectly tested using various statistical methods.

### 2.2. Participants and data source

The research data were primarily derived from 3 distinct projects. Genome-wide association study (GWAS) data for 91 inflammatory cytokines (ICs) were obtained from 11 cohorts comprising 14,824 individuals of European ancestry.^[16]^ Summary statistics for 1091 plasma metabolites and 309 metabolite ratios associated with human disease progression were acquired from the GWAS catalog (https://www.ebi.ac.uk/gwas/studies/GCST90199621-902010209), collected from the Canadian Longitudinal Study on Aging cohort with 8299 randomly selected participants.^[17]^ Publicly accessible GWAS summary data for TAA were sourced from the FinnGen database (https://storage.googleapis.com/finngen-public-data-r10/summary_stats/finngen_R10_I9_THAORTANEUR.gz), representing the most recent TAA-related dataset, comprising 385,857 participants (3880 cases and 381,977 controls).

All GWAS data included in this study for MR analysis are of European ancestry to minimize potential bias due to demographic heterogeneity. As the present MR study was based on previously collected and published data, no additional ethics approval was required.

### 2.3. Selection and validation of genetic instruments

Due to the limited number of single nucleotide polymorphisms (SNPs) extracted at the genome-wide significance threshold of *P* < 5 × 10^−8^, we relaxed the criteria to *P* < 1 × 10^−5^ for instrumental variable selection, an approach previously applied in MR studies.^[18,19]^ To evaluate the independence of SNPs against linkage disequilibrium, we adopted the PLINK clumping method, excluding SNPs with quality control parameters: *r*^2^ ≥ 0.001, clumping window ≤10,000 kb.^[20]^

*R*^2^ and *F*-statistics were calculated to estimate the strength of instrumental variables, with *F*-statistics >10 considered robust enough to guard against weak instrument bias.^[21]^ The *R*^2^ and *F*-statistic of each SNP were calculated according to the formulae: *R*^2^ = 2 × EAF × (1 − EAF) × β^2^ and *F* = *R*^2^ × (N−*K*−1)/(*K* × [1 − *R*^2^]), where *R*^2^ represents the proportion of variance in exposures explained by the genetic instrument, *K* represents the number of SNPs, N represents the sample size, β is the estimated effect size of the SNP and EAF represents the effect allele frequency.^[22]^

Palindromic SNPs were discarded due to uncertainty in aligning these SNPs in the same direction for exposure and outcome in the GWAS data. Exposure-related SNPs unavailable in the outcome GWAS were excluded. Subsequently, we harmonized remaining exposure and outcome SNPs and corrected incompatible SNPs to ensure that the effects of SNPs on the exposure and outcome correspond to the same allele.

### 2.4. MR statistical analysis

For our primary analysis, we employed the inverse-variance weighted (IVW) method, which combines the Wald ratios of each SNP into an overall weighted effect, under the assumption that all genetic variants in the analysis are valid IVs. To examine the reliability and consistency of the causal relationship between exposure and outcome, we performed several complementary MR analysis methods, including the weighted median method, MR Egger regression method, simple mode, and weighted mode.

We conducted sensitivity analyses to identify underlying pleiotropy and heterogeneity in MR estimates. The MR Egger intercept served as an indicator for horizontal pleiotropy.^[23]^ Additionally, we performed the Mendelian Randomized Polymorphism RESidual Sum and Outlier (MR-PRESSO) test to identify outlier SNPs that may have affected the causal estimates due to horizontal pleiotropy.^[24]^ We calculated the Cochrane *Q* test to examine heterogeneity among different genetic variations. To assess the consistency of the results, we conducted leave-one-out analysis, removing each SNP in turn to determine whether the overall estimates were disproportionately affected by any individual SNP.

All MR analyses were conducted using R software (version 4.4.0; R Foundation for Statistical Computing, University of Auckland, New Zealand) through the TwoSampleMR package (version 0.5.6) and MRPRESSO (version 1.0). We presented results as odds ratios (OR) with 95% confidence intervals (CI). Mediation effects were assessed using the product method, and mediation proportions were calculated according to the formula: (β1×β2)/β,^[25]^ where β represents the total effect size obtained from the primary analysis, β1 represents the effect size of ICs on mediators, and β2 represents the effect size of mediators on TAA.

### 2.5. Single cell sequencing data acquisition and processing

This study utilized the single-cell dataset GSE155468, comprising 3 normal samples and 6 TAA samples. Following stringent quality control of the raw data, we employed the Seurat package for data processing, encompassing normalization, variable feature selection, and batch effect correction via Harmony. Cell clustering was executed using the Louvain algorithm (resolution 0.5), and marker genes were identified using the FindAllMarkers function (minimum percentage expression 0.25, log-fold change threshold 0.5). Cell type annotation was based on established marker genes and augmented by the scMayoMap tool (Mayo Clinic, Rochester).

Differential expression analysis was performed using the DESeq2 model, with results visualized through volcano plots. Enrichment analysis was conducted using the ClusterGVis package (*P*-value threshold .05). The CellChat package was employed to construct cell–cell communication networks based on the human secretome database, with a minimum cell number threshold of 10.

The investigation focused on comparing gene expression patterns and cell–cell communication differences between normal and TAA conditions, with specific emphasis on key genes including C-C motif chemokine 20 (CCL20), CD40L receptor (CD40), C-X-C motif chemokine 10 (CXCL10), and fibroblast growth factor 5 (FGF5). Statistical significance was evaluated using the Wilcoxon rank-sum test. Results were visualized through diverse methods, including heatmaps, network diagrams, and violin plots, to elucidate the molecular mechanisms and cellular interactions in TAA. All analyses were executed in the R 4.4.0 environment, facilitating a comprehensive exploration of the molecular underpinnings and intercellular dynamics of TAA.

### 2.6. Protein–protein interaction (PPI) and identification of druggable proteins

To explore potential interactions between identified ICs, we constructed a PPI network using the Search Tool for the Retrieval of Interacting Genes database (https://string-db.org/). We further assessed whether the identified ICs could serve as potential therapeutic targets by searching for interactions between these ICs and drugs using The Drug Gene Interaction Database version 5.0 (https://www.dgidb.org). This database prioritizes potential druggable targets by integrating information from drug–gene interactions, gene function, and text mining.^[26]^ The drug-inflammatory cytokine interaction network was visualized using Cytoscape (version 3.10.2).

## 3. Results

### 3.1. Causal effects of inflammatory cytokines on TAA

**Figure 2.**
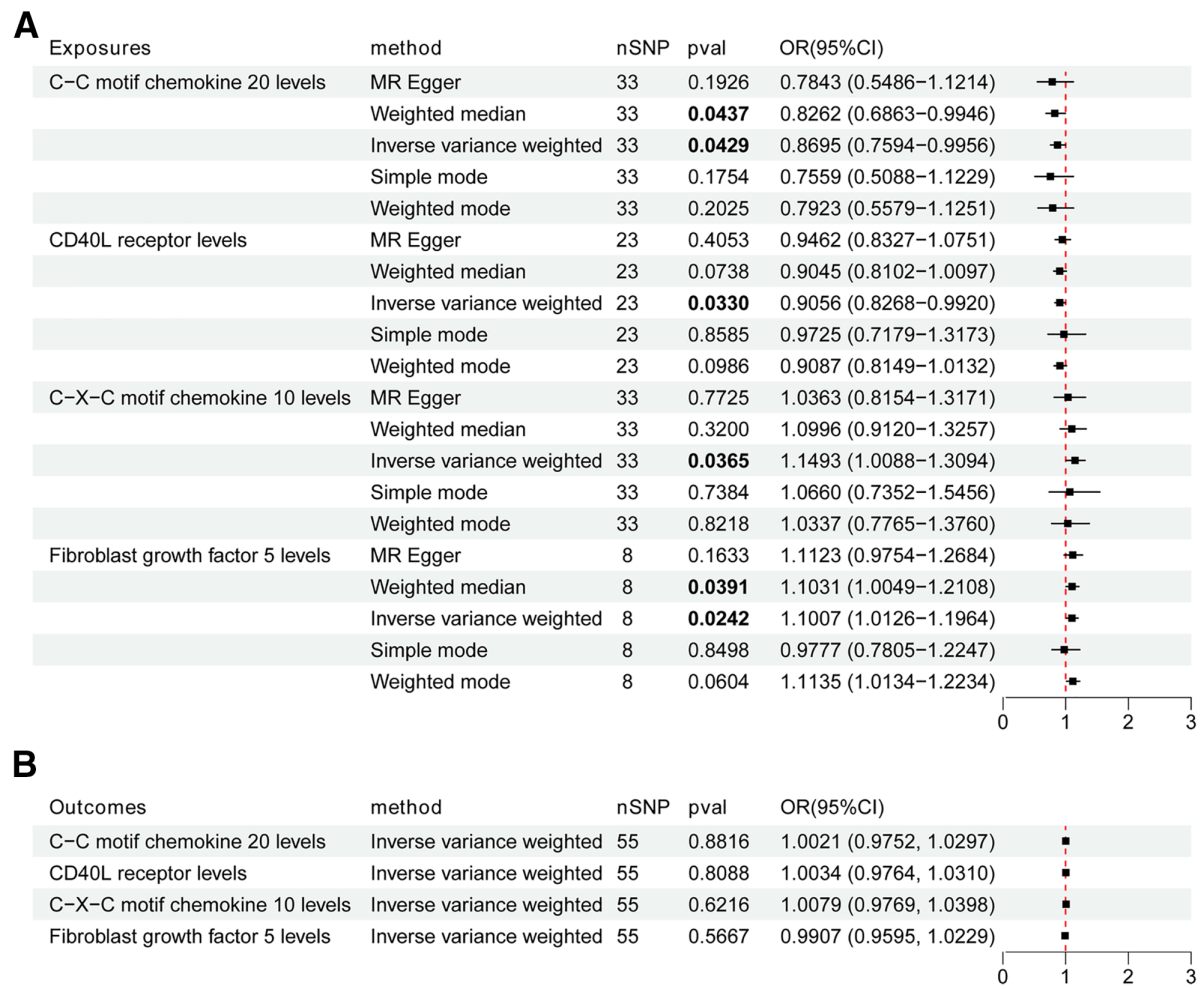
MR analysis of the relationship between ICs and TAA. (A) MR analysis estimates the causal effect of ICs on TAA. (B) MR analysis estimates the causal effect of TAA on ICs. CI = confidence interval; ICs = inflammatory cytokines; MR = Mendelian randomization; nSNP = number of single nucleotide polymorphism; OR = odds ratio; TAA = thoracic aortic aneurysm; *P* values <.05 are marked bold.

The primary IVW analysis revealed that CXCL10 (OR = 1.149, 95% CI = 1.009–1.309, *P* = .037) and FGF5 (OR = 1.101, 95% CI = 1.013–1.196, *P* = .024) significantly increased the risk of TAA. Conversely, CCL20 (OR = 0.870, 95% CI = 0.759–0.996, *P* = .043) and CD40 (OR = 0.906, 95% CI = 0.827–0.992, *P* = .033) demonstrated a protective effect against TAA (Fig. 2A). Detailed information on these 4 ICs is presented in Table S1, Supplemental Digital Content, https://links.lww.com/MD/R329. Complementary MR analyses, including MR Egger, weighted median, weighted mode, and simple mode, corroborated these findings for all ICs, with exception of the simple mode method for FGF5 (Table S2, Supplemental Digital Content, https://links.lww.com/MD/R329). Scatter plots and forest plots of SNP effect sizes for ICs and TAA (Figs. S1 and S2, Supplemental Digital Content, https://links.lww.com/MD/R328, respectively) further support these results.

Sensitivity analyses revealed no evidence of heterogeneity or pleiotropy (*P* > .05). The MR-PRESSO global test confirmed the absence of pleiotropy (*P* > .05, Table S3, Supplemental Digital Content, https://links.lww.com/MD/R329). Leave-one-out sensitivity analysis demonstrated that no individual SNP disproportionately influenced the overall effects of ICs on TAA (Fig. S3, Supplemental Digital Content, https://links.lww.com/MD/R328). The symmetrical funnel plot further validated the absence of horizontal pleiotropy (Fig. S4, Supplemental Digital Content, https://links.lww.com/MD/R328).

Reverse MR analysis indicated no causal relationship between TAA and the 4 selected ICs (*P* > .05, Fig. 2B). Detailed information on TAA-associated SNPs, MR results, and sensitivity analyses are provided in Tables S4–S6, Supplemental Digital Content, https://links.lww.com/MD/R329.

### 3.2. Causal effects of plasma metabolites on TAA

**Figure 3.**
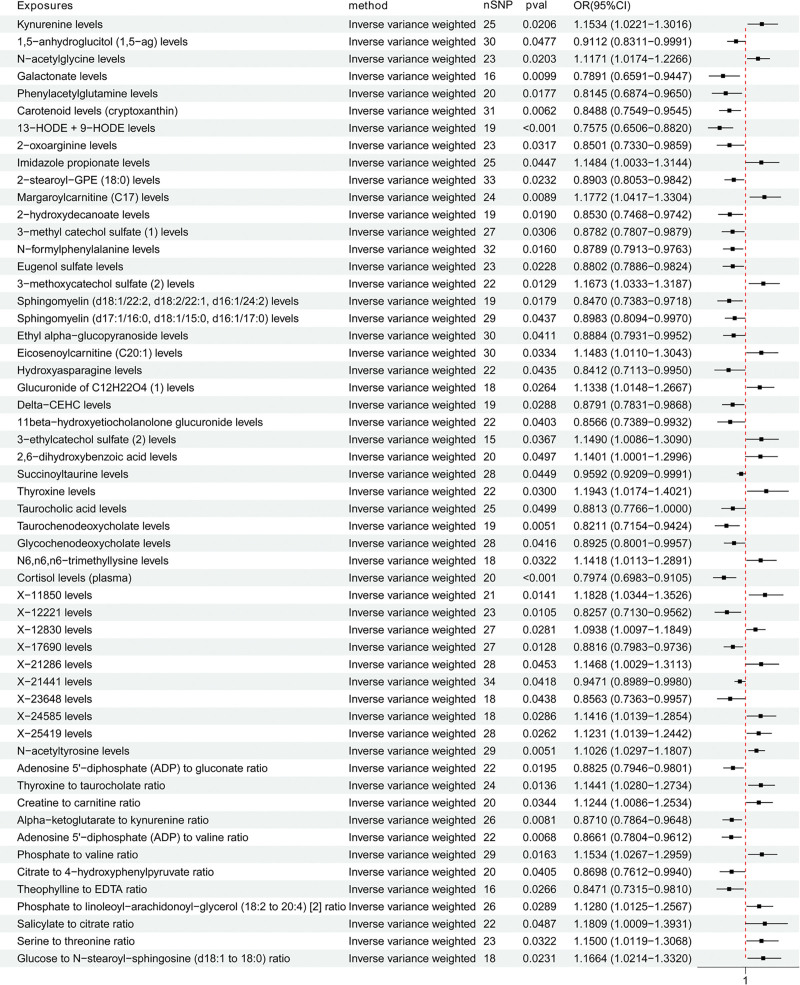
Forest plot to visualize the causal effects of the plasma metabolites on TAA (inverse variance weighted results). CI = confidence interval; nSNP = number of single nucleotide polymorphism; OR = odds ratio; TAA = thoracic aortic aneurysm.

The primary IVW analysis of 1091 plasma metabolites and 309 metabolite ratios identified 43 plasma metabolites and 12 metabolite ratios significantly associated with TAA risk (*P* < .05, Fig. 3). Notably, thyroxine emerged as the metabolite most likely to increase TAA risk (OR = 1.194, 95% CI: 1.017–1.402), while 13-HODE + 9-HODE demonstrated the strongest protective effect (OR = 0.758, 95% CI: 0.651–0.882). Results from complementary MR analyses are detailed in Table S7, Supplemental Digital Content, https://links.lww.com/MD/R329, with heterogeneity, pleiotropy, and MR-PRESSO global test analyses presented in Table S8, Supplemental Digital Content, https://links.lww.com/MD/R329.

Sensitivity analyses led to the exclusion of 2 metabolite ratios (salicylate to citrate ratio, *P*_pleiotropy_ = .019; serine to threonine ratio, *P*_pleiotropy_ = .013) due to evidence of pleiotropy. The remaining 43 plasma metabolites and 10 metabolite ratios were selected as potential mediators for further MR analysis to explore the influences of ICs on these possible mediators.

### 3.3. Causal effects of inflammatory cytokines on plasma metabolites

**Figure 4.**
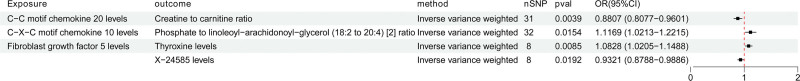
Forest plot to visualize the causal effects of ICs on plasma metabolites (inverse variance weighted results). CI = confidence interval; ICs = inflammatory cytokines; nSNP = number of single nucleotide polymorphism; OR = odds ratio.

MR inverse-variance weighted analysis, using SNPs of 4 ICs (CXCL10, FGF5, CCL20, and CD40) as exposure factors and 43 plasma metabolites and 10 metabolite ratios as outcome factors, revealed significant causal relationships. CXCL10 positively influenced the phosphate to linoleoyl-arachidonoyl-glycerol (18:2–20:4) [2] ratio (OR = 1.117, 95% CI: 1.021–1.222). FGF-5 showed a positive correlation with thyroxine (OR = 1.083, 95% CI: 1.021–1.149) and a negative association with X-24585 (OR = 0.932, 95% CI: 0.879–0.989). CCL20 negatively affected the creatine to carnitine ratio (OR = 0.881, 95% CI: 0.808–0.960). Detailed results are presented in Figure 4.

Complementary MR analyses (MR Egger, weighted median, weighted mode, and simple mode) corroborated these findings (Table S9, Supplemental Digital Content, https://links.lww.com/MD/R329). Scatter plots and forest plots of SNP effect sizes are provided in Figures S5 and S6, Supplemental Digital Content, https://links.lww.com/MD/R328, respectively. Analyses of heterogeneity, pleiotropy, and MR-PRESSO global test results are presented in Table S10, Supplemental Digital Content, https://links.lww.com/MD/R329. Leave-one-out sensitivity analysis and funnel plots are shown in Figures S7 and S8, Supplemental Digital Content, https://links.lww.com/MD/R328, respectively.

### 3.4. Mediation proportion

Based on MR analyses, 2 plasma metabolites and 2 metabolite ratios were identified as potential mediators. Their total effect size, mediation effect size, and mediation proportion were calculated (Table 1). In the FGF5 and TAA association, thyroxine (mediation proportion: 14.58%) and X-24585 were identified as mediators. The phosphate to linoleoyl-arachidonoyl-glycerol (18:2 to 20:4) [2] ratio (mediation proportion: 9.35%) mediated the relationship between CXCL10 and TAA. The creatine to carnitine ratio (mediation proportion: 10.71%) mediated the impact of CCL20 on TAA.

**Table 1:** Results of mediation proportion in two-step MR analysis.

Exposure	Mediator	Outcome	Total effect size β (95% CI)	The effect of exposure on mediator β1 (95% CI)	The effect of mediator on outcome β2 (95% CI)	Mediation effect size β1×β2	Mediated proportion (%)
CCL20	Creatine to carnitine ratio	TAA	−0.140 (−0.275 to −0.004)	−0.127 (−0.214 to −0.041)	0.117 (0.009 to 0.226)	−0.015	10.71
CXCL10	Phosphate to linoleoyl-arachidonoyl-glycerol (18:2 to 20:4) [2] ratio	TAA	0.139 (0.009 to 0.270)	0.111 (0.021 to 0.200)	0.120 (0.012 to 0.228)	0.013	9.35
FGF-5	Thyroxine	TAA	0.096 (0.013 to 0.179)	0.080 (0.020 to 0.139)	0.178 (0.017 to 0.338)	0.014	14.58
FGF-5	X-24585	TAA	0.096 (0.013 to 0.179)	−0.070 (−0.129 to −0.011)	0.132 (0.014 to 0.251)	−0.009	/

CCL20 = C-C motif chemokine 20, CD40 = CD40L receptor, CI = confidence interval, CXCL10 = C-X-C motif chemokine 10, FGF-5 = Fibroblast growth factor 5, MR = Mendelian randomization, TAA = thoracic aortic aneurysm.

### 3.5. Quality control of single-cell sequencing results

We performed comprehensive quality control and analysis of the single-cell RNA sequencing (scRNA-seq) data. Using the Seurat package, we conducted preliminary filtering on the raw data, selecting cells with gene counts between 200 and 4000 and UMI counts <20,000. We excluded cells with mitochondrial gene expression exceeding 10% to eliminate potential dead cells or cellular debris, and filtered out cells with hemoglobin gene expression surpassing 1% to mitigate red blood cell contamination. The results before (Fig. S9A, Supplemental Digital Content, https://links.lww.com/MD/R328) and after (Fig. S9B, Supplemental Digital Content, https://links.lww.com/MD/R328) quality control were visualized using violin plots, illustrating the distribution of gene counts (nFeature_RNA), RNA counts (nCount_RNA), mitochondrial gene expression percentage (percent.mt), ribosomal gene expression percentage (percent.rb), and hemoglobin gene expression percentage (percent.HB) for each sample.

Following data normalization, we selected the 3000 most variable genes for downstream analysis. Dimensionality reduction was performed through principal component analysis, with the optimal number of principal components determined using the elbow plot method. To eliminate potential batch effects, we applied the Harmony algorithm for data integration, ensuring comparability across different samples. The integrated data were then subjected to nonlinear dimensionality reduction to generate Uniform Manifold Approximation and Projection (UMAP) plots. The results before processing are shown in Figure S9C, Supplemental Digital Content, https://links.lww.com/MD/R328, while the integrated data are presented in Figure S9D, Supplemental Digital Content, https://links.lww.com/MD/R328.

Finally, we conducted a gene expression variability analysis, displaying the mean expression levels and standard deviations of genes in a scatter plot (Fig. S9E, Supplemental Digital Content, https://links.lww.com/MD/R328). This analysis revealed a series of genes exhibiting high variability under TAA conditions, including CCL20, SPP1, IL1B, and CXCL1, providing valuable directions for in-depth investigation of the molecular mechanisms underlying TAA.

### 3.6. Single-cell sample annotation and gene distribution differences

**Figure 5.**
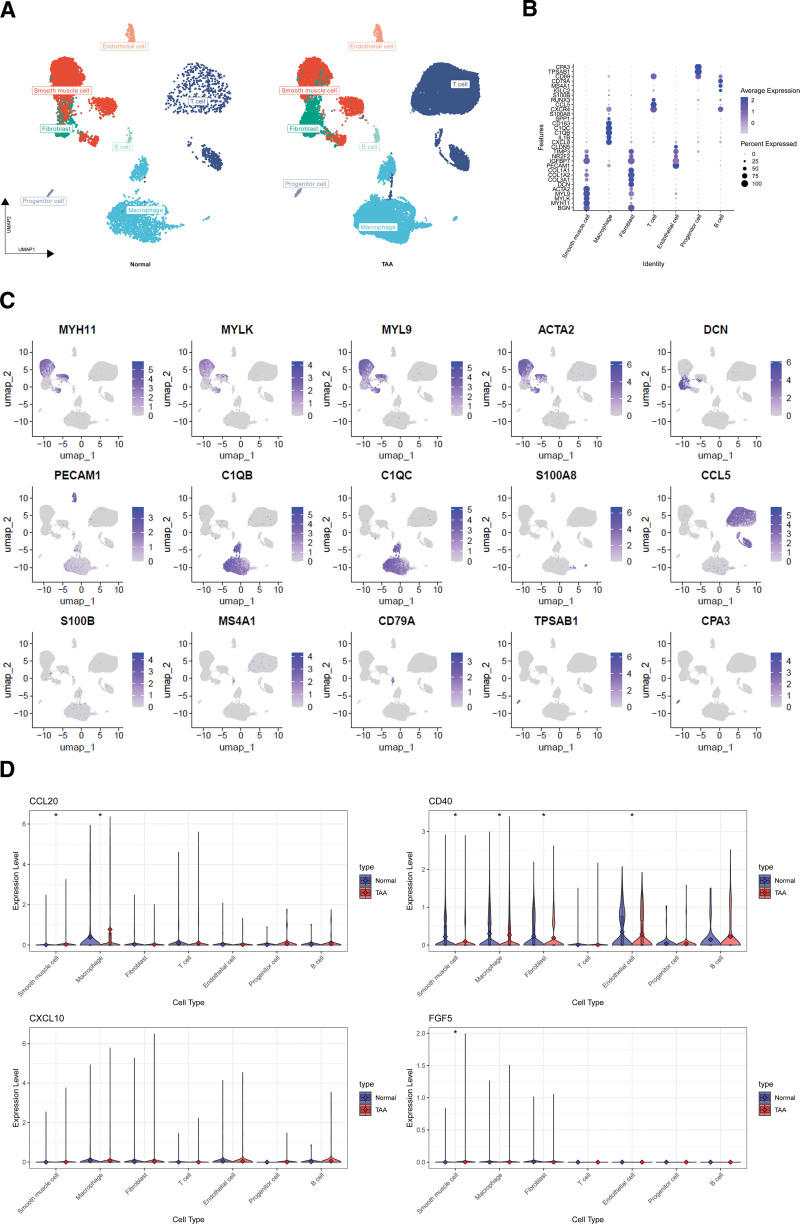
Single-cell RNA sequencing analysis reveals cellular heterogeneity and differential gene expression in normal and TAA tissues. (A) UMAP visualization of cell types in normal and TAA aortic tissues. (B) Dot plot showing expression patterns of key marker genes across identified cell types. (C) Feature plots demonstrating the expression distribution of important cell type-specific genes. (D) Violin plots comparing the expression of Mendelian randomization-identified TAA-associated genes (CCL20, CD40, CXCL10, and FGF5) between normal and TAA samples across different cell types. CCL20 = C-C motif chemokine 20; CD40 = CD40L receptor; CXCL10 = C-X-C motif chemokine 10; FGF5 = fibroblast growth factor 5; TAA = thoracic aortic aneurysm, UMAP = Uniform Manifold Approximation and Projection.

We applied the Louvain algorithm for cell clustering on the rigorously quality-controlled data, with a resolution of 0.5 to optimize clustering granularity and biological relevance. Characteristic genes for each cluster were identified using the FindAllMarkers function, with a minimum expression percentage of 0.25 and a log fold change threshold of 0.5. Cell clusters were precisely annotated by integrating scMayoMap results and literature findings. The UMAP dimensionality reduction visualization illustrates the distribution of various cell types from normal and TAA aortas, including smooth muscle cells, endothelial cells, fibroblasts, and diverse immune cells (Fig. 5A). To validate our annotations, we generated a dot plot depicting expression patterns and intensities of key marker genes across cell types (Fig. 5B).

Feature plots were employed to visualize the expression distribution of crucial genes in the UMAP space, including smooth muscle cell markers (MYH11, MYLK, and MYL9), endothelial cell marker (PECAM1), and T cell markers (CCL5 and RUNX3) (Fig. 5C). We focused on 4 genes previously associated with TAA through MR studies: CCL20, CD40, CXCL10, and FGF5. Violin plots were used to compare the expression differences of these genes across cell types in normal and TAA samples (Fig. 5D). Results revealed significant upregulation of CCL20 in smooth muscle cells and macrophages of TAA samples; CD40 was significantly downregulated in multiple cell types of TAA samples; CXCL10 showed no statistically significant differences; and FGF5, despite low overall expression, exhibited a slight increase in smooth muscle cells of TAA samples.

### 3.7. Molecular functional pathway differences in single cells

Following our analysis of gene distribution differences, we conducted a comprehensive examination of cell–cell communication networks in normal and TAA samples using the CellChat package. Our focus centered on CCL, CXCL, and FGF signaling pathways. By comparing cellular communication patterns between normal and TAA samples, we identified relevant changes in intercellular interactions under disease conditions.

**Figure 6.**
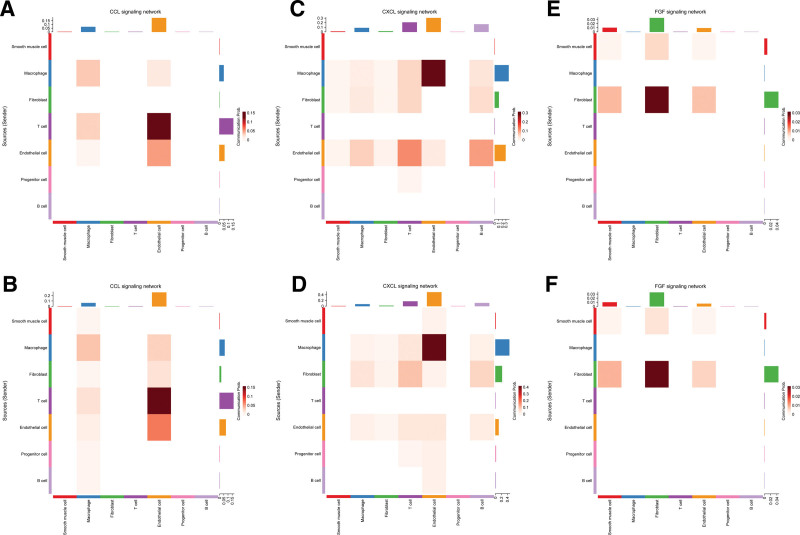
Comparative analysis of cell–cell communication networks in normal and TAA tissues. (A) CCL signaling network in normal aortic tissue. (B) CCL signaling network in TAA tissue. (C) CXCL signaling network in normal aortic tissue. (D) CXCL signaling network in TAA tissue. (E) FGF signaling network in normal aortic tissue. (F) FGF signaling network in TAA tissue. CCL = C-C motif chemokine; CXCL = C-X-C motif chemokine; FGF = fibroblast growth factor; TAA = thoracic aortic aneurysm.

While no significant overall functional differences were observed between the signaling networks, subtle alterations were noted. The CCL signaling network revealed more active signal interactions involving macrophages in TAA samples, suggesting an enhanced immune response (Fig. 6A and B). In the CXCL signaling network, TAA samples exhibited weakened T cell signal reception and enhanced endothelial cell signal reception (Fig. 6C and D). This change underscores the potentially crucial role of CXCL signaling in the pathological process of TAA, particularly in coordinating immune cell recruitment and vascular inflammatory responses.

The FGF signaling network also displayed subtle alterations between normal and TAA samples, although these changes were less pronounced compared to the CCL and CXCL networks (Fig. 6E and F). These findings collectively provide insights into the complex cellular communication dynamics underlying TAA pathogenesis and highlight potential targets for further investigation and intervention.

The observed changes in cell–cell communication networks offer valuable insights into the molecular mechanisms underlying TAA development and progression. Our results suggest that alterations in immune cell interactions and vascular signaling may play significant roles in the pathogenesis of TAA. These findings open avenues for future research and potential therapeutic strategies targeting these specific signaling pathways.

### 3.8. PPI and identification of druggable proteins

**Figure 7.**
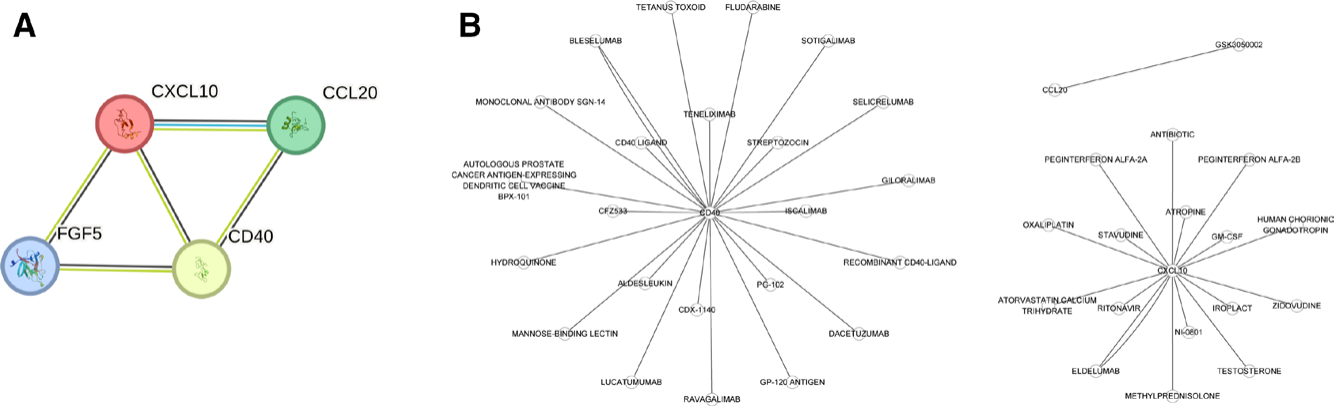
PPI and identification of druggable proteins. (A) PPI analysis displayed the connection among the 4 selected ICs. (B) The analysis of drug targets revealed 17 drug molecules targeting CXCL10, 24 targeting CD40 and one potential drug molecule targeting CCL20. CCL20 = C-C motif chemokine 20, CD40 = CD40L receptor, CXCL10 = C-X-C motif chemokine 10, ICs: inflammatory cytokines, PPI = protein–protein interaction.

We conducted PPI analysis to elucidate the relationships among the identified causal ICs (Fig. 7A). In our evaluation of druggable proteins, we identified 3 ICs (CXCL10, CCL20, and CD40L) as potential drug targets. Our analysis revealed 17 drug molecules targeting CXCL10 and 24 targeting CD40. For CCL20, we identified only one potential drug molecule (Fig. 7B). Comprehensive details of these findings are presented in Table S11, Supplemental Digital Content, https://links.lww.com/MD/R329.

## 4. Discussion

Based on MR analysis and single-cell RNA sequencing data, we conducted an in-depth investigation into the potential roles of key inflammatory factors in the pathogenesis of TAA. Our study employed a bidirectional two-sample MR analysis to evaluate the causal association between ICs and TAA. We included 91 ICs, 1400 plasma metabolites and TAA GWAS summary statistics from 385,857 participants. Our results indicated that higher genetically determined levels of CXCL10 and FGF-5 could increase the risk of TAA, while increases in CCL20 or CD40 might reduce this risk.

Furthermore, through two-step MR analysis, we discovered 2 plasma metabolites and 2 metabolite ratios (thyroxine, linoleoyl-arachidonoyl-glycerol (18:2 to 20:4) [2] ratio, creatine to carnitine ratio, and X-24585) that potentially act as intermediate molecules transmitting the pathogenic effects of ICs. Single-cell data further elucidated the cell-specific manifestations of these findings. Identification of drug targets prioritized 3 protein biomarkers with potential as therapeutic targets for TAA.

The role of ICs and plasma metabolites in the occurrence and progression of TAA has long been a focus of attention. Previous research has implicated CXCL10 in various cardiovascular diseases, including atherosclerosis, aneurysm formation, and myocardial infarction.^[27–29]^ Clinical studies have shown that patients with TAA exhibit significantly elevated circulating CXCL10 levels compared to controls.^[30]^ Based on these observational researches, our MR analysis further suggested that CXCL10 could increase the risk of TAA, validating the causality between CXCL10 and TAA.

To investigate possible downstream pathways of CXCL10, we employed plasma metabolites (the main component in plasma) as mediators and found that the phosphate to linoleoyl-arachidonoyl-glycerol ratio was affected by CXCL10. Emerging evidence indicates a link between disturbances in phosphate homeostasis and cardiovascular disease. Phosphate levels are associated with ejection fraction, and hypophosphatemia has been implicated in the development of heart failure and cardiac arrhythmias.^[31,32]^ The Coronary Artery Risk Development in Young Adults study demonstrated an association between high serum phosphate levels and vascular calcification in individuals with normal kidney function.^[33]^ Linoleoyl-arachidonoyl-glycerol has also been reported to increase the risk of coronary heart disease.^[34]^ Our findings suggest that CXCL10 may play a role in TAA occurrence via an increase in the phosphate to linoleoyl-arachidonoyl-glycerol ratio.

CXCL10 belongs to the CXC chemokine family, exerting its biological effects mainly via binding to CXCR3.^[35,36]^ In this study, cell communication network analysis indicated changes in CXCL signaling networks, potentially involving immune cell recruitment and vascular inflammatory response regulation. CXCL10 and CXCR3 expression have been reported to be related to the recruitment of CXCR3^+^ T lymphocytes secreting IFNγ, correlating with intimal expansion and outward arterial remodeling, which results in matrix degradation.^[37]^ However, CXCL10 showed no significant expression differences between TAA and normal samples in our scRNA-seq analysis, suggesting its effects may be more prominent at the protein level or during specific disease stages.

Our study indicated that FGF5 could increase the risk of TAA and its expression in smooth muscle cells of TAA samples was increased, although the underlying mechanism remains unclear. Previous research has shown that FGF5 is involved in myocardial wall thickness at end diastole in the apical anterior segment and heart failure susceptibility induced by hypertension.^[38]^ One study demonstrated that the expression level of FGF5 in sepsis heart was decreased and FGF5 overexpression could attenuate lipopolysaccharide-induced cardiac injury via the inhibition of CaMKII-mediated pyroptotic signaling.^[39]^ Another study suggested that human aortic endothelial cells have greater angiogenic potential through FGF5 upregulation and could be a compatible endothelial cell type to achieve robust angiogenesis.^[40]^ Additionally, we discovered that FGF5 could increase the level of thyroxine, potentially further increasing the risk of TAA. As is known, smooth muscle cell phenotype switching, degradation of elastic fibers, and extracellular matrix remodeling are the main causes of TAA formation.^[41]^ Thyroxine, as one of the thyroid hormones, has been reported to be involved in the formation of atheromatous plaques, thinning and rupture of the elastic lamellae, changes in thickness of the tunica media, and alterations in the density of elastic fibers, which may contribute to the occurrence of TAA.^[42]^

There is a close relationship between CCL20 and aortic disease. Plasma CCL20 expression is increased in abdominal aortic aneurysm (AAA) and could be used as a biomarker of AAA with high sensitivity.^[43]^ Another study showed that CCL20 was highly expressed in AAA patients and mouse models. Overexpression of CCL20 could restore the effect of transcription factor TCF3 on the decreased expression of MMP9 and MMP2, inhibition of fibrosis and elastin degradation in mouse models.^[44]^ In addition, scRNA-seq analysis revealed that the proportion of macrophages in acute type A aortic dissection tissues (24.51%) was significantly higher than that in normal tissues (13.69%), and CCL20 was expressed more intensively in macrophages in acute type A aortic dissection tissue than in those in normal tissue.^[45]^ Single-cell data in this study consistently displayed the upregulation of CCL20 in macrophages and smooth muscle cells in TAA samples. Interestingly, however, a negative causality between CCL20 and TAA was illustrated by the MR analysis, which contradicted the scRNA-seq analysis results. This may reflect the complexity of the disease process and the multifaceted roles of CCL20 at different stages of disease. MR analysis revealed that low CCL20 levels induced TAA, suggesting that the presence of low CCL20 precedes TAA. Nevertheless, the scRNA-seq analysis reflected the phenomenon that followed TAA formation. Based on the above, we could speculate that high CCL20 levels represent a reactive increase to exert a reparative function during the development of TAA. Prior MR analysis indicated that CD40 level was negatively associated with the risk of TAA.^[46]^ Consistently, we found that CD40 could decrease the risk of TAA and CD40 was significantly downregulated across multiple cell types in TAA samples. However, no mediator in the plasma metabolites was found, which indicated either direct regulation or the involvement of other mediators between CD40 and TAA.

scRNA-seq analysis also revealed changes in cellular heterogeneity during TAA development. In TAA samples, we observed significant alterations in cellular composition, particularly a decrease in the proportion of smooth muscle cells and an increase in immune cells (such as macrophages and T cells), reflecting tissue remodeling and enhanced inflammatory responses. The reduction in smooth muscle cells may lead to decreased vascular wall strength. The increase and activation of immune cells may promote TAA development through sustained inflammatory responses. The identification of druggable proteins, cell-specific expression patterns, and functional changes provided important clues for understanding the specific roles of inflammatory factors in TAA development, while also highlighting the potential value of targeting specific cell types for the treatment of TAA.

The use of biomarkers in clinical applications is becoming increasingly practical, providing crucial support for early diagnosis, personalized treatment, and disease prognosis assessment. Detecting biomarkers usually requires advanced equipment such as PCR, gene sequencing, and immunoassay devices. Imaging equipment and portable biosensors are also used to gather accurate data. Some of the equipment is already functioning in hospitals at various levels. To effectively use biomarkers, a network system integrating data collection, analysis, and processing is necessary. This system should utilize cloud platforms, AI algorithms, and big data analytics to monitor patient health in real time and support personalized treatment. Additionally, establishing an efficient data integration platform and ensuring data privacy protection are key to achieving this goal. As technology keeps advancing, the clinical application of biomarkers is poised to transform medical diagnosis and therapeutic strategies.

Although several studies have sought to elucidate the relationship between inflammation and TAA, our study is the first to comprehensively investigate the causal relationship between ICs and TAA using a two-sample, two-step MR design, which was based on genetic instruments selected from the largest current dataset of TAA and the latest summary-level data of 91 inflammatory cytokines. The use of bidirectional MR in our study minimized bias caused by reverse causation and confounders, which are the main limitations of traditional observational studies. Our analysis of mediators further explored the possible pathophysiological changes in cytokine-induced TAA. Our findings may have clinical significance as they demonstrate reliable results across various MR methods. The application of several sensitivity analyses verified the robustness of the MR results.

However, several limitations of this study warrant attention. First, the examined GWASs were primarily conducted in individuals of European ancestry, which may limit the generalizability of our findings to other ethnicities. Second, while aneurysms can be located in the ascending aorta, descending aorta and aortic arch, we were unable to investigate associations between ICs and TAA in these subpopulations due to the lack of individual-level data. Third, completely excluding the influence of potential pleiotropy is challenging in any MR study. Despite employing multiple sensitivity approaches, some residual pleiotropic effects may still exist. Lastly, while this study identified possible mediators, the underlying mechanisms require further investigation.

## 5. Conclusions

This research identified that CXCL10 and FGF5 increased the risk of TAA, while CCL20 and CD40 decreased the risk, providing new insights into the etiology of TAA and promising targets for the development of therapeutic interventions. Additionally, several plasma metabolites and metabolite ratios were found to mediate the causal pathways between ICs and TAA. Intervention targeting these factors may be beneficial for TAA prevention. Further experimental and clinical studies are needed to evaluate the utility and efficacy of these candidates and to validate our current findings. This work lays a foundation for future research into potential preventive and therapeutic strategies for TAA, emphasizing the importance of inflammatory pathways in its pathogenesis.

## Author contributions

**Conceptualization:** Yueheng Liu, Rui Tang.

**Data curation:** Yueheng Liu, Rui Tang, Fengming Tian.

**Formal analysis:** Yueheng Liu, Tianxiang Gu.

**Funding acquisition:** Yitan Fang, Tianxiang Gu.

**Investigation:** Yueheng Liu.

**Methodology:** Yueheng Liu, Rui Tang, Yitan Fang, Fengming Tian, Tianxiang Gu.

**Project administration:** Yueheng Liu, Rui Tang, Yitan Fang, Tianxiang Gu.

**Resources:** Yitan Fang, Tianxiang Gu.

**Software:** Yueheng Liu, Yitan Fang, Fengming Tian, Tianxiang Gu.

**Supervision:** Yueheng Liu, Rui Tang, Fengming Tian, Tianxiang Gu.

**Validation:** Yueheng Liu, Rui Tang, Fengming Tian.

**Visualization:** Yueheng Liu, Yitan Fang, Fengming Tian.

**Writing – original draft:** Yueheng Liu, Rui Tang, Yitan Fang, Fengming Tian, Tianxiang Gu.

**Writing – review & editing:** Yueheng Liu, Yitan Fang, Fengming Tian, Tianxiang Gu.

## Supplementary Material




