# Supplementary material for: Inflammatory cytokines mediate thoracic aortic aneurysm formation via plasma metabolites: A two-step Mendelian randomization and single cell sequencing-based investigation
Source: Medicine (Baltimore). 2026 Feb 6;105(6):e47561. doi: 10.1097/MD.0000000000047561 (PMC12885723; doi:10.1097/MD.0000000000047561)

Supplementary Figure S1. Scatter plots of the causal effect of ICs on TAA risk. The slopes of each line represent the causal association for each method. A, Scatter plot of the causal effect of C-C motif chemokine 20 on TAA risk. B, Scatter plot of the causal effect of CD40L receptor on TAA risk. C, Scatter plot of the causal effect of C-X-C motif chemokine 10 on TAA risk. D. Scatter plot of the causal effect of fibroblast growth factor 5 on TAA risk. ICs: inflammatory cytokines; TAA: thoracic aortic aneurysm.

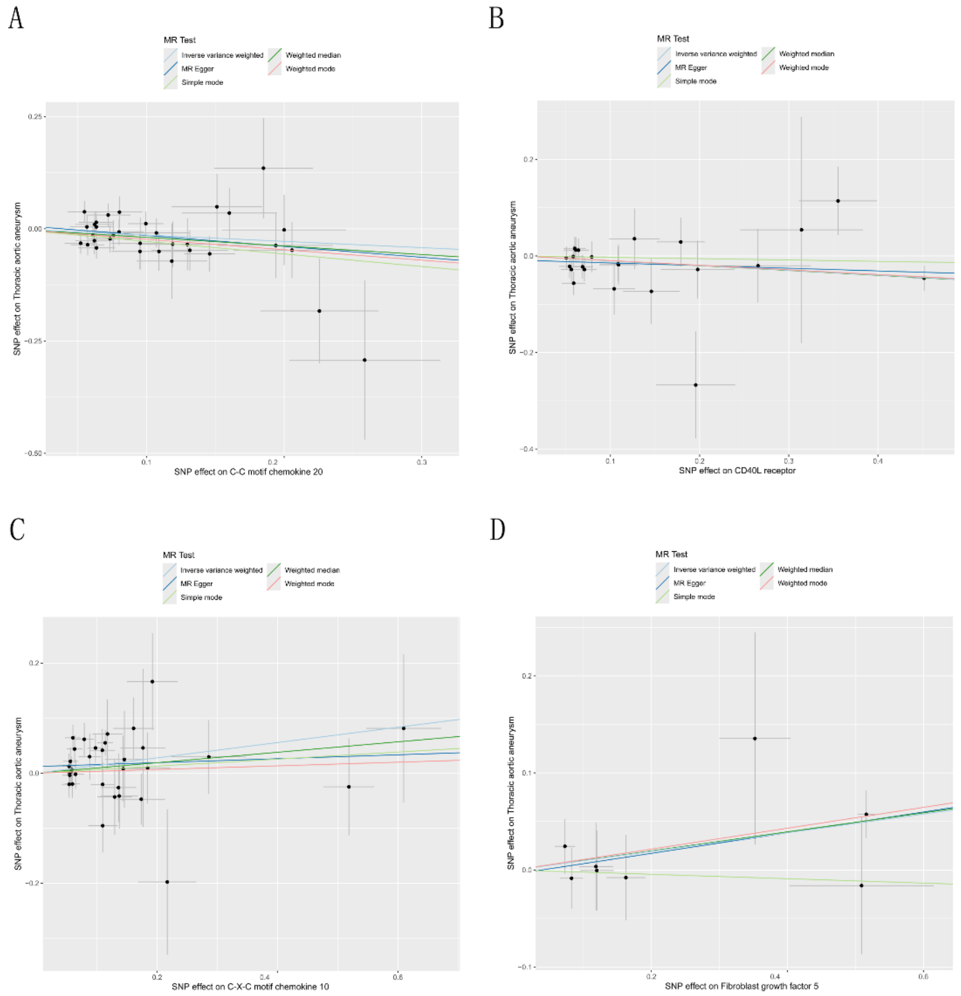

Supplementary Figure S2. Forest plots of MR analysis for ICs in TAA. A, Forest plots of MR analysis for C-C motif chemokine 20. B, Forest plots of MR analysis for CD40L receptor. C, Forest plots of MR analysis for C-X-C motif chemokine 10. D, Forest plots of MR analysis for fibroblast growth factor 5. MR: Mendelian randomization; ICs: inflammatory cytokines; TAA: thoracic aortic aneurysm.

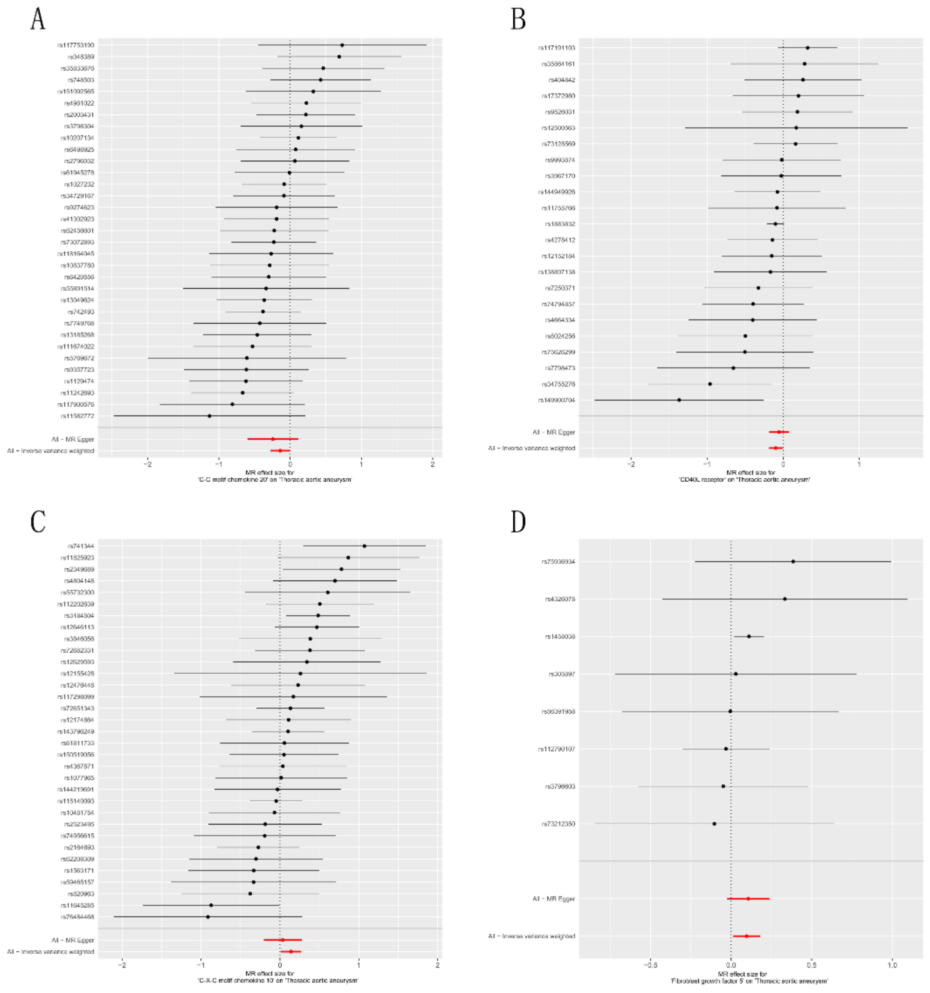

Supplementary Figure S3. Leave-one-out analysis of MR analysis for the association between ICs and TAA. A, Leave-one-out analysis for C-C motif chemokine 20. B, Leave-one-out analysis for CD40L receptor. C, Leave-one-out analysis for C-X-C motif chemokine 10. D, Leave-one-out analysis for fibroblast growth factor 5. MR: Mendelian randomization; ICs: inflammatory cytokines; TAA: thoracic aortic aneurysm.

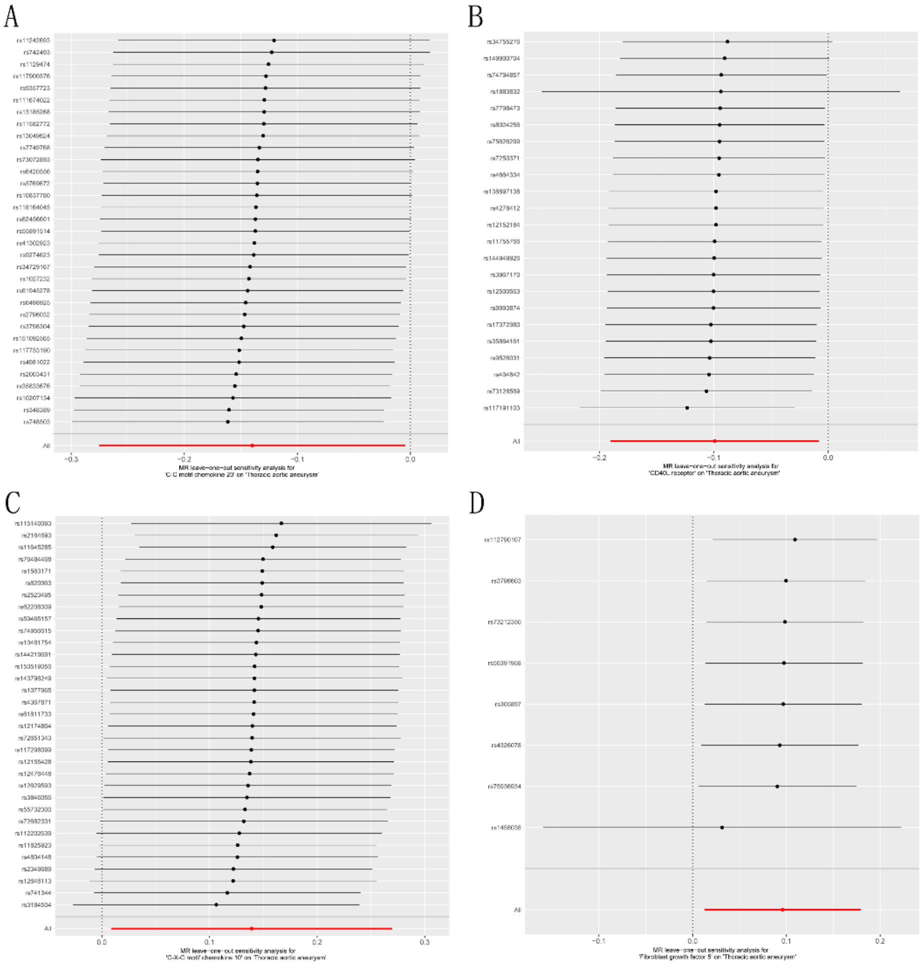

Supplementary Figure S4. Funnel plots of MR analysis for the association between ICs and TAA. A, Funnel plot for C-C motif chemokine 20. B, Funnel plot for CD40L receptor. C, Funnel plot for C-X-C motif chemokine 10. D, Funnel plot for Fibroblast growth factor 5. MR: Mendelian randomization; ICs: inflammatory cytokines; TAA: thoracic aortic aneurysm.

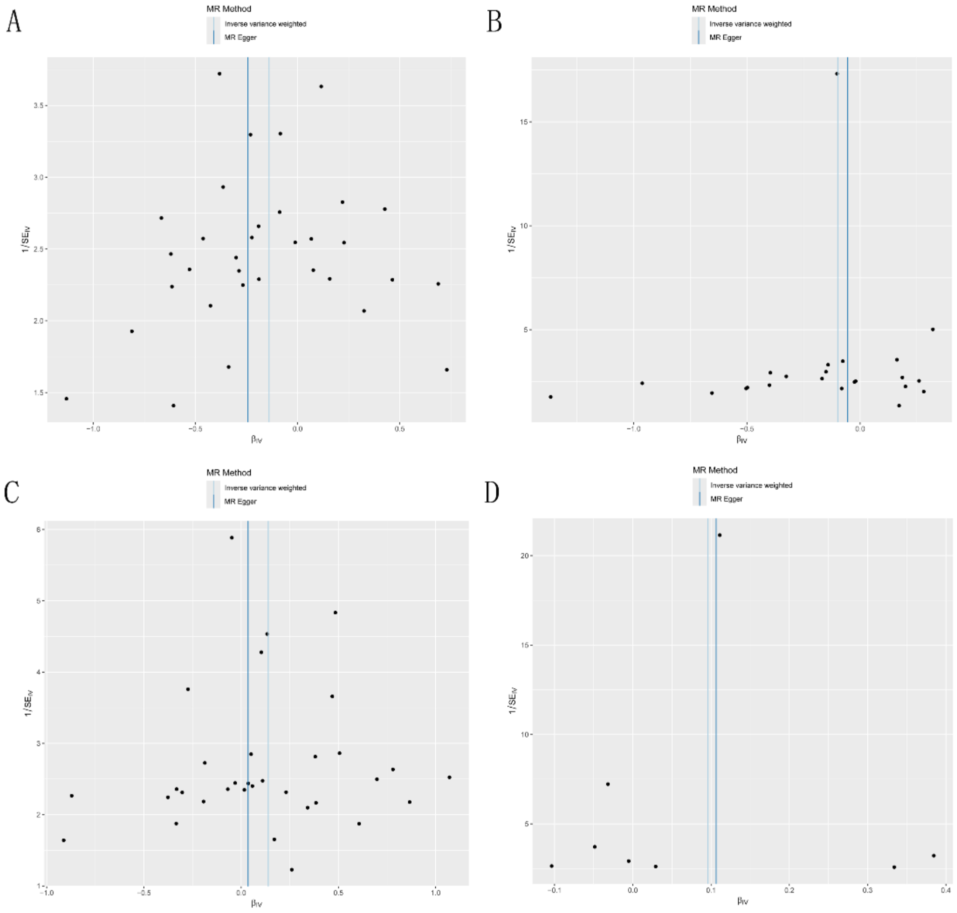

Supplementary Figure S5. Scatter plot of the causal effect of ICs on plasma metabolites. The slopes of each line represent the causal association for each method. A, Scatter plot of the causal effect of C-C motif chemokine 20 on creatine to carnitine ratio. B, Scatter plot of the causal effect of C-X-C motif chemokine 10 on phosphate to linoleoyl-arachidonoyl-glycerol (18:2 to 20:4) [2] ratio. C, Scatter plot of the causal effect of fibroblast growth factor 5 on thyroxine. D. Scatter plot of the causal effect of fibroblast growth factor 5 on X-24585. ICs: inflammatory cytokines.

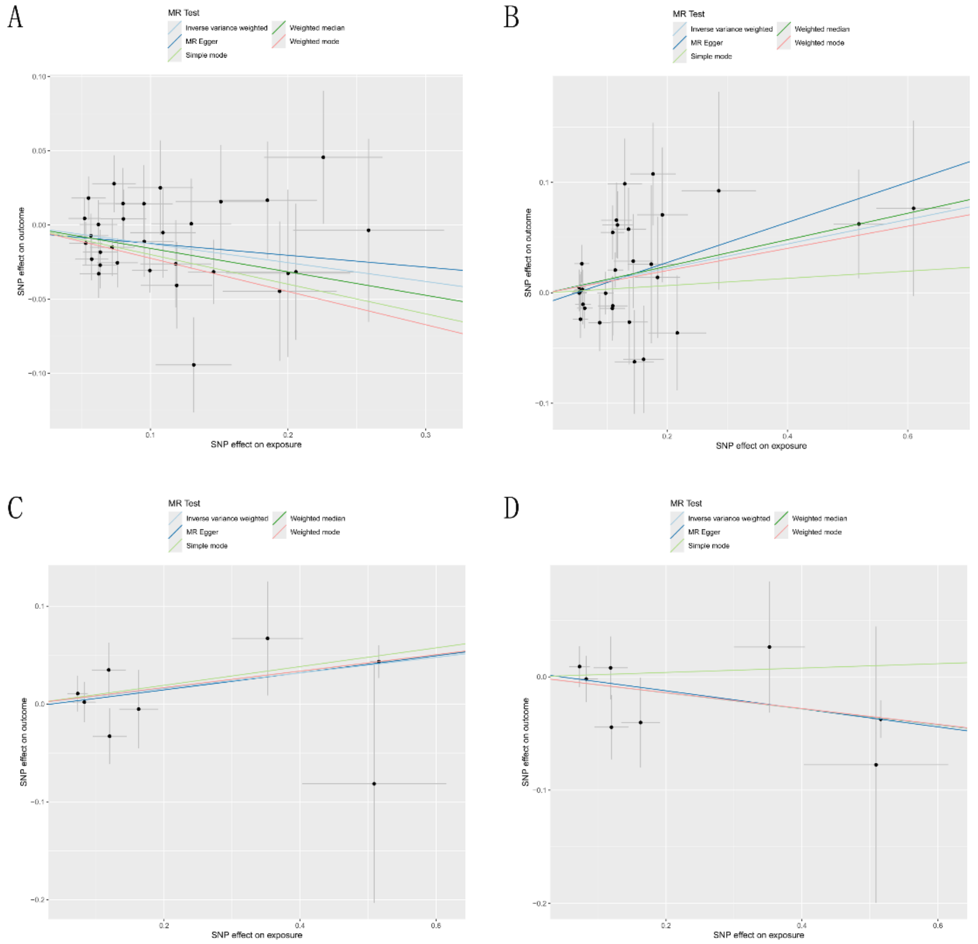

Supplementary Figure S6. Forest plots of MR analysis of the relationship between ICs and plasma metabolites. A, Forest plot of the relationship between C-C motif chemokine 20 and creatine to carnitine ratio. B, Forest plot of the relationship between C-X-C motif chemokine 10 and phosphate to linoleoyl-arachidonoyl-glycerol (18:2 to 20:4) [2] ratio. C, Forest plot of the relationship between fibroblast growth factor 5 and thyroxine. D, Forest plot of the relationship between fibroblast growth factor 5 and X-24585. MR: Mendelian randomization; ICs: inflammatory cytokines.

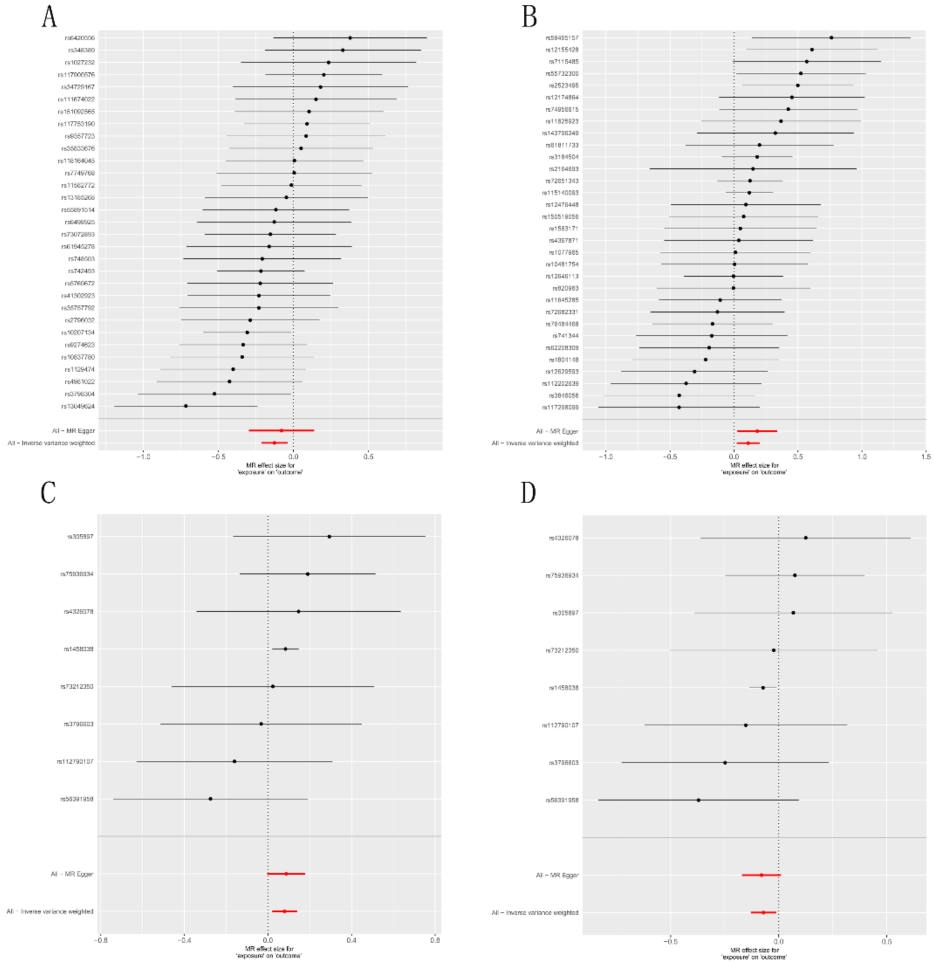

Supplementary Figure S7. Leave-one-out analysis of MR for the association between ICs and plasma metabolites. A, Leave-one-out analysis for the association between C-C motif chemokine 20 and creatine to carnitine ratio. B, Leave-one-out analysis for the relationship between C-X-C motif chemokine 10 and phosphate to linoleoyl-arachidonoyl-glycerol (18:2 to 20:4) [2] ratio. C, Leave-one-out analysis for the relationship between fibroblast growth factor 5 and thyroxine. D, Leave-one-out analysis for the relationship between fibroblast growth factor 5 and X-24585. MR: Mendelian randomization; ICs: inflammatory cytokines.

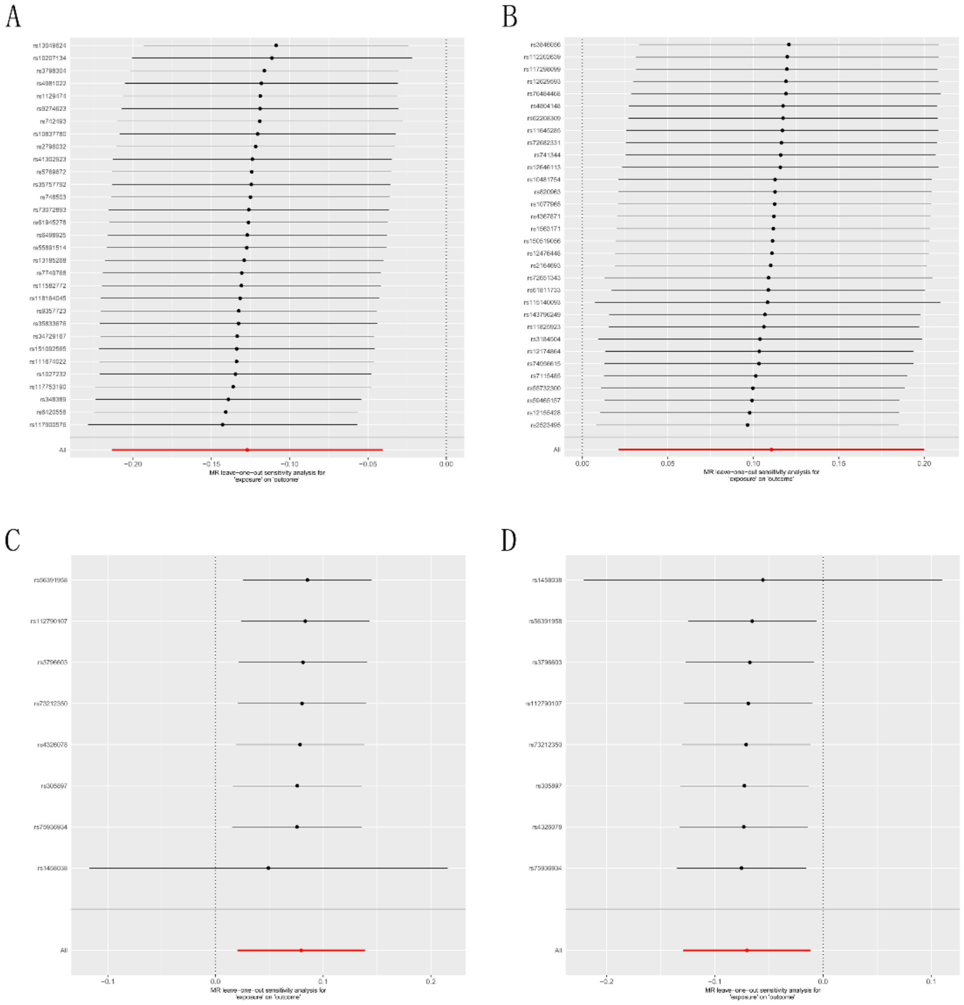

Supplementary Figure S8. Funnel plots of MR analysis for the association between ICs and plasma metabolites. A, Funnel plot for the association between C-C motif chemokine 20 and creatine to carnitine ratio. B, Funnel plot for the relationship between C-X-C motif chemokine 10 and phosphate to linoleoyl-arachidonoyl-glycerol (18:2 to 20:4) [2] ratio. C, Funnel plot for the relationship between fibroblast growth factor 5 and thyroxine. D, Funnel plot for the relationship between fibroblast growth factor 5 and X-24585. MR: Mendelian randomization; ICs: inflammatory cytokines.

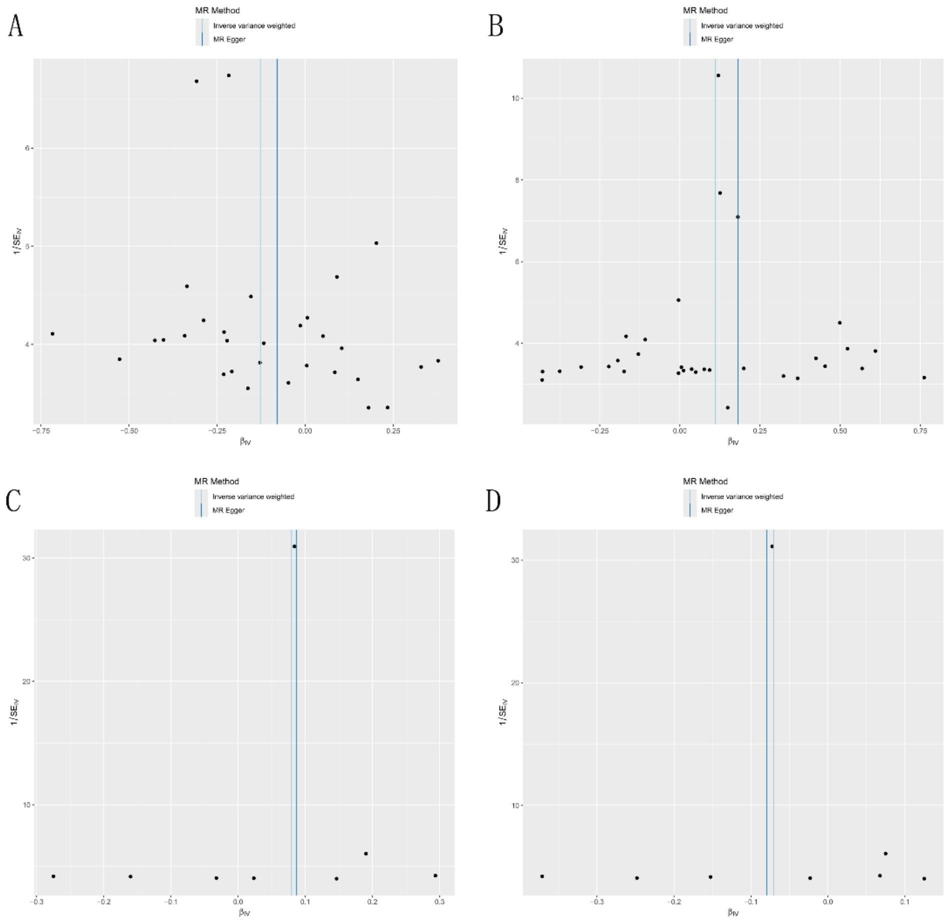

Supplementary Figure S9. Quality control and processing of single-cell RNA sequencing data in TAA study. A, Violin plots showing quality control metrics before filtering. B, Violin plots showing quality control metrics after filtering. C, UMAP visualization of cells before batch effect correction. D, UMAP visualization of cells after Harmony integration. E, Scatter plot of gene expression variability, highlighting highly variable genes in TAA condition.

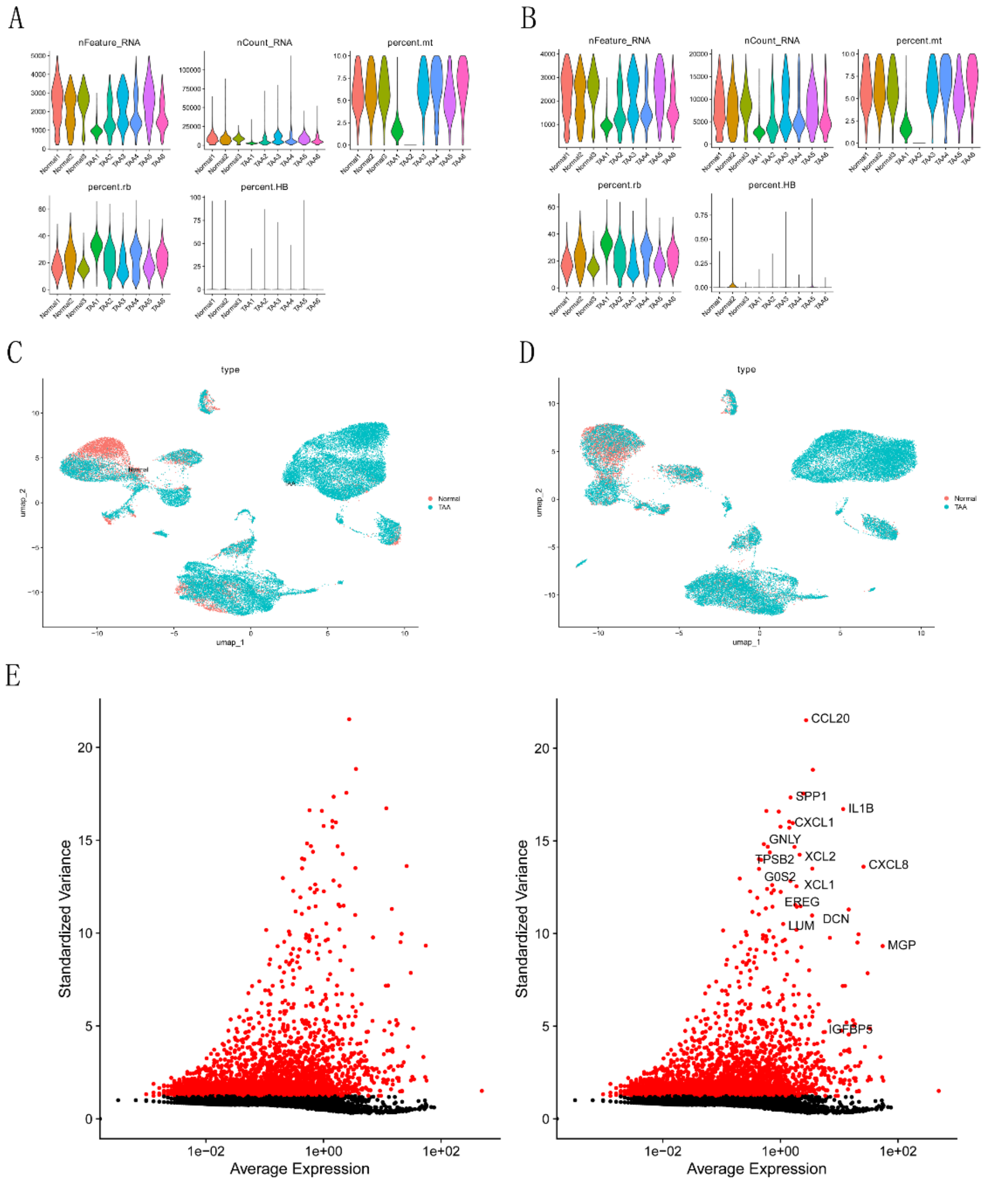

Supplement: Supplementary file 2 [file medi-105-e47561-s002.pdf]
